# Sex-specific association between low oral doses of cannabidiol (CBD) and plasma concentration of anandamide (AEA), N-palmitoylethanolamine (PEA) and N-oleoylethanolamine (OEA) in healthy occasional cannabis users

**DOI:** 10.1186/s42238-025-00356-x

**Published:** 2026-01-09

**Authors:** Anita Abboud, Lucy Ann Chester, Francois-Olivier Hébert, Didier Jutras-Aswad

**Affiliations:** 1 Department of Psychiatry and Addictology, Université de Montréal, Montréal, Québec Canada; 2 Research Center, CHUM (CRCHUM), 900 St-Denis Street, Viger Tower, 5Th Floor, room R05.740, Montreal, QC H2X 1P1 Canada

**Keywords:** Cannabidiol, Anandamide, Palmitoylethanolamide, Oleoylethanolamide, Acute effects, Crossover design

## Abstract

**Background:**

Cannabidiol (CBD), a phytocannabinoid produced by *Cannabis sativa*, is widely consumed and interacts with components of the endocannabinoid system, including enzymes and receptors, through indirect and complex mechanisms. However, how CBD influences endogenous cannabinoids such as anandamide (AEA), and related N-acylethanolamines like N-palmitoylethanolamine (PEA) and N-oleoylethanolamine (OEA), remains poorly understood. This study investigates the acute impact of marketed CBD doses on the plasmatic levels of these signaling lipids in occasional cannabis users, addressing a critical gap in understanding the biological effects of low-dose CBD in non-therapeutic contexts.

**Methods:**

In a triple-blind, placebo-controlled, randomized crossover trial, 70 healthy volunteers received ten sequences of four oral CBD doses (20, 50, 100, 200 mg) and placebo. Blood was sampled at baseline and five timepoints post-dose. Plasma AEA, PEA, and OEA were quantified by LC–MS, and dose–response assessed with linear mixed-effects models on plasma concentrations (model 1) and Area Under the Curve to increase (AUCi, model 2), including participant ID (nested in sequence) as random effect, and visit, sequence, sex, and baseline levels as fixed effects.

**Results:**

Model 2 revealed a significant effect of CBD dose on AUCi of PEA and OEA, but not AEA. Pairwise comparisons showed that placebo was associated with significantly higher AUCi values than the 50 mg dose (*p* < 0.05; moderate effect sizes), and trended higher than the 200 mg dose (*p* < 0.10; small-to-moderate effect sizes). No differences were observed for other dose contrasts. Importantly, sex emerged as a significant factor: sub-group analyses indicated that these reductions in AUCi were driven by female participants, with lower PEA and OEA exposure confirmed at both 50 mg and 200 mg (only PEA) compared to placebo. No corresponding effects were observed in males. All plasma levels decreased overall throughout each visit, at every dose of CBD and placebo.

**Conclusions:**

This study revealed that among healthy adults who consume cannabis occasionally, low-dose oral CBD formulations were able to significantly decrease the cumulative plasma levels of PEA and OEA in a female-specific fashion, confirming the importance of sex-differences in cannabinoid response, and emphasizing the relevance of a personalized approach to cannabis consumption and its effects, as well as public health messaging.

**Trial registration:**

(ClinicalTrials.gov, registration: NCT05407285, Registration date: 2022–06-02).

**Supplementary Information:**

The online version contains supplementary material available at 10.1186/s42238-025-00356-x.

## Introduction

In North America, cannabidiol (CBD)-based products have gained popularity since cannabis legalization, with approximately 16% to 22% of cannabis users in Canada and the United States reporting the use of CBD-containing products (Québec and statistique du. Institut de la statistique du Québec. Enquête québécoise sur Le cannabis 2023; Goodman et al. 2022). Although cannabidiol (CBD) is often described as non-psychoactive, this phytocannabinoid has rapidly gained popularity as an over-the-counter supplement. More than half of CBD users (Goodman et al. 2022; Les effets bénéfiques du cannabidiol : conclusions hâtives ou faits avérés - Innofibre 2025) report using it for its purported sleep-inducing (Ranum et al. 2023; Wang et al. 2024), analgesic (Mohammed et al. 2024; Cásedas et al. 2024), neuroprotective (Jantas et al. 2024; Aychman et al. 2023; Tambe et al. 2023), and pro-cognitive (Solowij et al. 2018; Lees et al. 2023; Bomfim et al. 2023) effects. A decentralized, observational study on US adults conducted by Kaufmann et al. also revealed that CBD is primarily used for chronic pain (27%), mental health (22%), general health and wellness (20%) and sleep disorders (10%) (Kaufmann et al. 2023) outside of clinical or prescription use.

These reported effects of CBD may be partly attributed to its diverse mechanisms of action, as it interacts with over 65 pharmacological targets, including those within the endocannabinoid system (ECS) (Reggio 2010). The ECS, which includes the main endogenous cannabinoids N-arachidonoylethanolamide (anandamide; AEA) and 2-arachidonoylglycerol (2-AG), plays a vital role in maintaining homeostasis by regulating key physiological functions such as motor activity, pain, stress, sleep, appetite, and hunger sensation (Calvino 2021).

Previous studies have shown that CBD has the potential to influence the endocannabinoid system by modulating levels of AEA, among other crucial functions, in the regulation of emotions and stress by promoting the feeling of happiness and relaxation (Bluett et al. 2014; Kwee et al. 2023; Bisogno et al. 2001; Couttas et al. 2023, 2024; Leweke et al. 2012; Hua et al. 2023). AEA belongs to a broader class of bioactive lipids known as N-acylethanolamines (NAEs), which also includes its congeners N-palmitoylethanolamine (PEA) and N-oleoylethanolamine (OEA) (Iannotti et al. 2016; Ueda et al. 2010), which exhibit anti-inflammatory and metabolic effects independently of cannabinoid receptor activation (Hutch et al. 2020; Petrosino and Di Marzo 2017; Fu et al. 2003; Paterniti et al. 2013). One possible mechanistic explanation involves CBD competing with NAEs for binding to fatty acid-binding proteins (FABPs), which transport them to fatty acid amide hydrolase (FAAH) for degradation, leading to modulation of NAE levels (Elmes et al. 2015; Kaczocha et al. 2012). However, whether CBD exerts such effects through the modulation of PEA and OEA levels remains poorly understood in humans (Couttas et al. 2024; Di Marzo and Piscitelli 2015; Kaczocha et al. 2012). Studies have also tried to look at the effect of CBD on 2-AG levels in human studies, but no significant changes were observed (Couttas et al. 2024; Chester et al. 2024).

Despite the ECS’s central role in physiological regulation, few clinical studies have evaluated CBD’s regulating impact on circulating endocannabinoid concentrations, especially NAEs. This represents a major limitation in understanding CBD’s mechanisms in humans. Studies in other pathological contexts have shown that ECS-related signaling lipids can be significantly altered, and that ECS components are highly expressed in reward-related neural circuits (Parsons and Hurd 2015). Moreover, genetic variations in ECS-related genes (e.g., CNR1, FAAH) are linked to traits like impulsivity and addiction vulnerability (Parsons and Hurd 2015). A systematic review published in 2024 found that AEA levels are consistently elevated in individuals with substance use disorders, while 2-AG levels are more variable (Elliott et al. 2025). In schizophrenia, CBD treatment has been associated with increased serum AEA levels, which correlated with symptom improvement, suggesting a therapeutic mechanism via AEA modulation (Leweke et al. 2012). Given these findings, NAEs may serve as sensitive indicators for CBD’s biological effects and could be studied to better understand CBD’s molecular action.

Regulatory processes under the ECS control can also be modulated by internal parameters, as biological sex may be an important factor influencing endocannabinoid signaling and the effects of cannabinoids. Evidence suggests sex-dimorphism in the endogenous cannabinoid system: a large cross-sectional study reported higher circulating levels of AEA, 2-AG, OEA, and PEA in males compared to females, with patterns that shift across the lifespan in relation to hormonal changes (Amir Hamzah et al. 2023). Preclinical and translational work also indicates sex differences in CB1 receptor density, signaling efficacy, and enzymatic activity involved in endocannabinoid metabolism (Forner-Piquer et al. 2024; Craft et al. 2013; López 2010; Blanton et al. 2021). Moreover, behavioral and subjective responses to cannabinoids often differ between males and females (Blanton et al. 2021; Rubino and Parolaro 2011). Yet, few human studies have directly examined whether CBD modulates NAEs in a sex-specific manner. This gap highlights the importance of considering sex as a potential moderator, which may help explain variability in findings and improve the generalizability of CBD research.

Considering the rising popularity of CBD products and the abundance of anecdotal reports from individuals using them in diverse contexts, there remains a significant gap in clinical data regarding how low, non-prescription doses of CBD influence the ECS. Most existing studies have focused on animal models (Bluett et al. 2014; Filippis et al. 2008), clinical populations (Hua et al. 2023; Lichenstein 2022), or have administered high doses of CBD (often exceeding 300 mg/day) (Couttas et al. 2024; Chester et al. 2024; Bloomfield et al. 2022) or have used CBD in combination with THC (Chester et al. 2024; Martin-Willett et al. 2024; Walter et al. 2013). These approaches do not reflect the real-world usage patterns observed in the general population. In the observational study of Kaufmann et al., the reported mean daily doses, ranged from 48 to 61 mg/day (Kaufmann et al. 2023). These results show that outside prescription use, reported CBD doses are significantly lower than those used in interventional studies’ products or in prescription formulations such as Epidiolex (between 5 to 20 mg/kg/day) (Dosing and Administration| EPIDIOLEX® (cannabidiol) 2025).

Importantly, healthy individuals who use cannabis occasionally represent a distinct and understudied population, even though they represent more than half of cannabis users in Canada (Lichenstein 2022; Chesney et al. 2020; Québec and statistique du. Institut de la statistique du Québec. Enquête québécoise sur Le cannabis 2023; Canada 2024). These individuals are more likely to use CBD products recreationally or for general wellness rather than for managing a diagnosed medical condition (Goodman et al. 2022; Kaufmann et al. 2023; Canada 2024). They tend to seek low-psychoactive cannabis products from legal retail stores, where product labeling is often limited and little information is provided about the specific effects of these compounds (Canada 2021). This represents a large group of consumers who ingest active cannabinoids in non-medical contexts with minimal guidance on dosage-effect relationships. Investigating the biological effects of CBD in this population is therefore critical, not only to understand how marketed doses available in non-clinical settings interact with the endocannabinoid system and influence key lipid-derived signaling compounds, but also to generate robust scientific data from controlled environments. Such data are essential for helping cannabis users make informed purchasing decisions and for developing realistic, evidence-based public health messages.

The overall objective of the current study was to evaluate the acute biological effects of low-dose CBD (20 mg to 200 mg) versus placebo in healthy, occasional cannabis users. Specifically, we aimed to characterize the influence of CBD on plasmatic concentrations of AEA, PEA, and OEA, three NAEs implicated in emotional regulation, inflammation, and metabolic processes. We hypothesized that CBD administration would result in a significant, dose-dependent increase in the levels of these lipid compounds, with the 200 mg dose exerting the most pronounced effect. In addition, given evidence for sex-related variability in endocannabinoid signaling, we conducted exploratory analyses to assess whether biological sex moderates CBD’s effects on NAE levels. By addressing both dose–response and potential sex differences, this study seeked to bridge the gap between real-world CBD use and mechanistic understanding by examining peripheral NAE levels as an index of how low-dose CBD may modulate the ECS in a non-clinical population.

## Methods

### Trial design

This study is an exploratory analysis of a triple-blind, placebo-controlled, randomized, crossover trial that aimed to compare the acute behavioral and physiological effects of different oral low doses of synthetic CBD and placebo in healthy occasional cannabis users. The present study focuses on characterizing the influence of low-dose CBD on plasmatic concentrations of AEA, PEA, and OEA, based on samples collected during the parent trial. Eligible healthy occasional cannabis users were randomized to ten pre-established sequences of four doses of CBD (20 mg, 50 mg, 100 mg and 200 mg) and placebo (0 mg). The trial, which was conducted at *the Centre Hospitalier de l’Université de Montréal* (CHUM) Research Center, was approved by the CHUM research ethics committee and followed relevant ethical guidelines (Helsinki Declaration, International Standards of Good Clinical Practice, Tri-Council Policy Statement, Health Canada division 5 guidelines). All participants gave their written informed consent prior to enrollment. The trial protocol was registered at ClinicalTrials.gov (NCT05407285).

### Participants

A total of 71 healthy occasional cannabis users were recruited and randomized between 2022 and 2023 at the CHUM Research Center, of whom 70 received at least one dose of study drug. Recruitment was carried out through advertisements on social media, flyers, websites, and word of mouth. Interested volunteers completed a pre-screening questionnaire to determine general eligibility for the trial, based on their age, ability to communicate in French and/or English, if they had ever used cannabis in their life, if they were pregnant or planning to become pregnant, and if they were breastfeeding. Occasional cannabis users were defined as participants reporting using cannabis three days or less in the 28 days prior to enrollment, based on the definition provided by the *Institut National de Santé Publique du Québec* (INSPQ) (Consommation de cannabis chez la population générale (tiré de l’EQC) | INSPQ 2025). They were also considered ‘healthy’ if they did not present with any severe or unstable medical or psychiatric condition, immunodeficiency, or current substance use disorder (other than nicotine). Participants were required to meet the following key inclusion criteria: aged 21 to 65 years; having used cannabis three days or less in the 28 days prior to enrollment; able to provide signed informed consent; and willing to comply with study procedures, including abstaining from other cannabis products or drugs (except alcohol or nicotine) for seven days prior to study visits. Key exclusion criteria were: severe and/or unstable medical or psychiatric conditions, immunodeficiency, other substance use disorder (except nicotine), pregnancy (confirmed by urine pregnancy test) or plans to become pregnant, and recent medication use that may interact with cannabis.

### Research product

Study products contained pharmaceutically produced CBD, formulated as a THC-free (< 10 ppm) oral oil with an initial concentration of 100 mg/ml. Inactive ingredients include medium-chain triglyceride (MCT) oil and Vitamin E. The cannabinoid-free placebo was composed of inactive ingredients only (MCT oil and Vitamin E). To maintain blinding, a total of 2 mL of the study product was administered at each session. For the active products (doses of 20 mg, 50 mg, 100 mg, and 200 mg of CBD), the required volume of CBD oil was mixed with the placebo oil to reach a total of 2 mL, thus ensuring an identical taste, colour and texture among all study products. The study product was mixed and dispensed by the CR-CHUM pharmacy.

### Sample size

A target sample size of 70 participants was determined based on a power calculation for the trial’s primary outcome, i.e. peak pleasant drug effect. Using a factorial ANOVA model to compare the 200 mg dose with placebo in a balanced 5 × 5 Latin Square design, the calculation assumed α = 0.05, 80% power, and a medium effect size (Cohen’s d = 0.50) (Hayes and Bennett 1999; Pourhoseingholi et al. 2013). Allowing for an anticipated 10% dropout rate led to the final target of 70 participants.

### Randomization and blinding

At the first study visit, participants were randomly assigned to one of ten predetermined dose sequences based on a balanced 5 × 5 Latin square design. Investigators, participants, data analysts, and research staff responsible for administering the study product and assessing research outcomes remained blinded to the treatment dose, with only the dispensing pharmacist being unblinded.

### Procedure

Participants attended a total of five study visits, each separated by a washout period of at least seven days. This interval was chosen based on the estimated elimination half-life of oral CBD (ranging from ~ 18 to 32 h), ensuring sufficient time for systemic clearance and reducing the risk of pharmacological carryover between doses (Devinsky et al. 2014). Before receiving the study drug at each visit, participants underwent urine testing for cannabis, drug and alcohol use as well as pregnancy. If participants tested positive for any urine drug test, they were asked to reschedule the visit. An indwelling cannula was then inserted into the non-dominant arm to facilitate blood collection. Each visit started around 9 am, lasting approximately seven hours, and involved the completion of a dozen outcome assessments. A standardized high-fat meal (at least 55 g of fat) developed by certified nutritionists specifically for this study was provided immediately before product ingestion, followed by two low-fat snacks at the 120- and 360-min timepoints. Participants were permitted to consume tea or coffee with their snacks; however, smoking breaks were not allowed. Adverse events were collected during each study visit with the Systematic Assessment of Side Effects in Clinical Trials (SAFTEE) form.

### Outcome measures

The main outcome measures of the present paper correspond to the exploratory outcomes of the parent trial, specifically the plasma levels of AEA, PEA and OEA. Additional exploratory data collected in the main trial included pharmacokinetic data and genetic profiles, though these are not analyzed here.

For context, the primary outcome of the parent trial was the peak pleasant drug effect, assessed using a visual analog scale. Secondary outcomes included intoxication, dissociation, specific subjective effects, cognitive functioning, psychotic symptoms, anxiety, affect, and dose discrimination. A semi-structured interview at each session captured additional effects not identified through quantitative measures.

### Blood sampling and extraction procedure

Blood samples (10 ml each) were drawn at pre-ingestion (0 min; T0) and at 60, 120-, 210-, 300-, and 360-min post-ingestion during each visit (T60, T120, T210, T300 and T360, respectively). Due to budget limitations, AEA concentrations were assessed at baseline (T0), at the estimated peak CBD plasma concentration (T120) (Izgelov et al. 2020; Wheless et al. 2019; Millar et al. 2018), and at the end of the visit (T360), whereas PEA and OEA were measured at all six timepoints.

Samples were collected from the forearm using an indwelling cannula and immediately transferred into 4 ml EDTA tubes. Samples were centrifuged at 1600 g for 10 min at room temperature and plasma was transferred into pre-labeled 500 µl microtubes. Handling time was kept under 5 min, and all plasma samples were processed within 20 min. The microtubes were then stored in designated freezer boxes at −80 °C. Sample processing was performed by an external nursing unit following standard procedures, which do not include 4 °C centrifugation.

AEA, PEA and OEA plasma levels were determined by high performance liquid chromatography-tandem mass spectrometry (HPLC–MS/MS) at the Pharmacokinetics Core Facility at CR-CHUM. See Supplementary Methods S1 and S2 for full extraction procedures.

### Statistical analysis

All analyses were performed using R version 4.3.2 statistical software. Two linear mixed-effects models assessed the relationships between endocannabinoid levels and CBD doses, developed with the lme4 R package (Bates et al. 2015). Both models included participant ID nested within randomization sequence as a random effect and visit number, dosing sequence, sex and baseline plasma levels (T0) as fixed effects. Model 1 examined the association between CBD dose (categorical) and extreme endocannabinoid concentrations, defined as either Cmin or Cmax, depending on the average direction of change from baseline. Model 2 evaluated the effect of CBD dose on the Area Under the Curve (AUC) of endocannabinoid levels across visits, which reflects overall exposure. The AUC was calculated as the area under the curve with respect to increase (AUCi), in which the area between baseline levels (T0) and ground is removed from all subsequent time points, allowing control for baseline differences across participants (Pruessner et al. 2003). Both models were ran independently on AEA, PEA, and OEA.

Pairwise comparisons between estimated marginal means (emmeans R package, version 1.10.6) (Lenth and emmeans: Estimated Marginal Means, aka Least-Squares Means. 2017) were conducted to determine which CBD doses were significantly associated with changes in both outcomes. Models 1 and 2 provided the estimated marginal means for comparison between all CBD doses. Cohen’s d (effect size R package) (Ben-Shachar et al. 2020), 95% confidence intervals and p-values adjusted with the Tukey method for multiple comparisons were provided for these pairwise comparisons. *Post-hoc* exploratory subgroup analyses were also conducted for sex in both models to examine potential sex-specific effects of CBD.

All statistical tests were two-sided, with a 5% significance level. Model assumptions were checked with the DescTools (version 0.99.58) (Signorell 2025) and the car (Fox et al. 2025) R packages, and BoxCox data transformation (MASS package) (Venables and Ripley (2002) ensured normality. Multiplicity was accounted for using the Tukey adjustment approach. No imputation method was applied for missing data, as the proportion of missing data was minimal (i.e., less than 10%).

## Results

### Baseline data

Seventy out of seventy-one randomised participants received at least one dose of study drug. Of these, 68 participants completed all five visits of the study, with two dropouts due to adverse events (AEs) not related to study product. The median age of participants (with interquartile range, Q1–Q3) was 31.2 (21.4–65.7) years, with 54.3% assigned female at birth and 78.4% identifying as White. Median frequency of cannabis use in the past 28 days was 1 (1–1.75) day across the sample. Median baseline (T0) levels of AEA, PEA and OEA were 0.3261 (0.1201–0.8468) ng/ml, 1.613 (0.475–4.107) ng/ml and 1.357 (0.475–3.291) ng/ml, respectively. Additional baseline demographic characteristics are provided in Table 1.

**Table 1:** Demographics and AEA, PEA and OEA measurements at baseline

Baseline characteristics*		All participants (*N* = 70)
Age		31.20 (21.44–65.74)
Sex		
	Male	32 (45.7)
	Female	38 (54.3)
Gender		
	Man	33 (47.1)
	Woman	36 (51.4)
	Non-binary	1 (1.4)
Ethnic Group**		
	Caucasian/White	55 (78.6)
	South Asian (e.g. Indian/Pakistani)	3 (4.3)
	Other Asian	4 (5.7)
	Hispanic/Latin	6 (8.6)
	North African/Middle Easter	4 (5.7)
	Black: African	1 (1.4)
	Black: Caribbean	1 (1.4)
	Metis	1 (1.4)
BMI		
	Male	24.46 (17.67–32.43)
	Female	22.86 (16.37–32.45)
Education		
	Secondary school diploma	2 (2.9)
	Diploma or certificate from a trade school or vocational program (DVS)	10 (14.3)
	University undergraduate degree (certificate, minor, major, bachelor's)	35 (50.0)
	Master's, doctorate or graduate degree	14 (20.0)
	Other	9 (12.9)
Days of alcohol use in the last 28 days		3.0 (0.0–28.0)
Days of other substance use in the last 28 days		0.0 (0.0–7.0)
Days of cannabis use in the last 28 days		1.0 (0.0–3.0)
Baseline endocannabinoid levels (ng/ml)		
	AEA	0.3261 (0.1201–0.8468)
	PEA	1.613 (0.475–4.107)
	OEA	1.357 (0.475–3.291)

*AEA* Anandamide, *BMI* Body mass index, *OEA* Oleoylethanolamide, *PEA* Palmitoylethanolamide

^*^Categorical variables are reported as N(%), continuous variables are reported as Median (Min–Max)

^**^Participants could select more than one ethnic group

### Plasma levels throughout visit timepoints

**Fig. 1:**
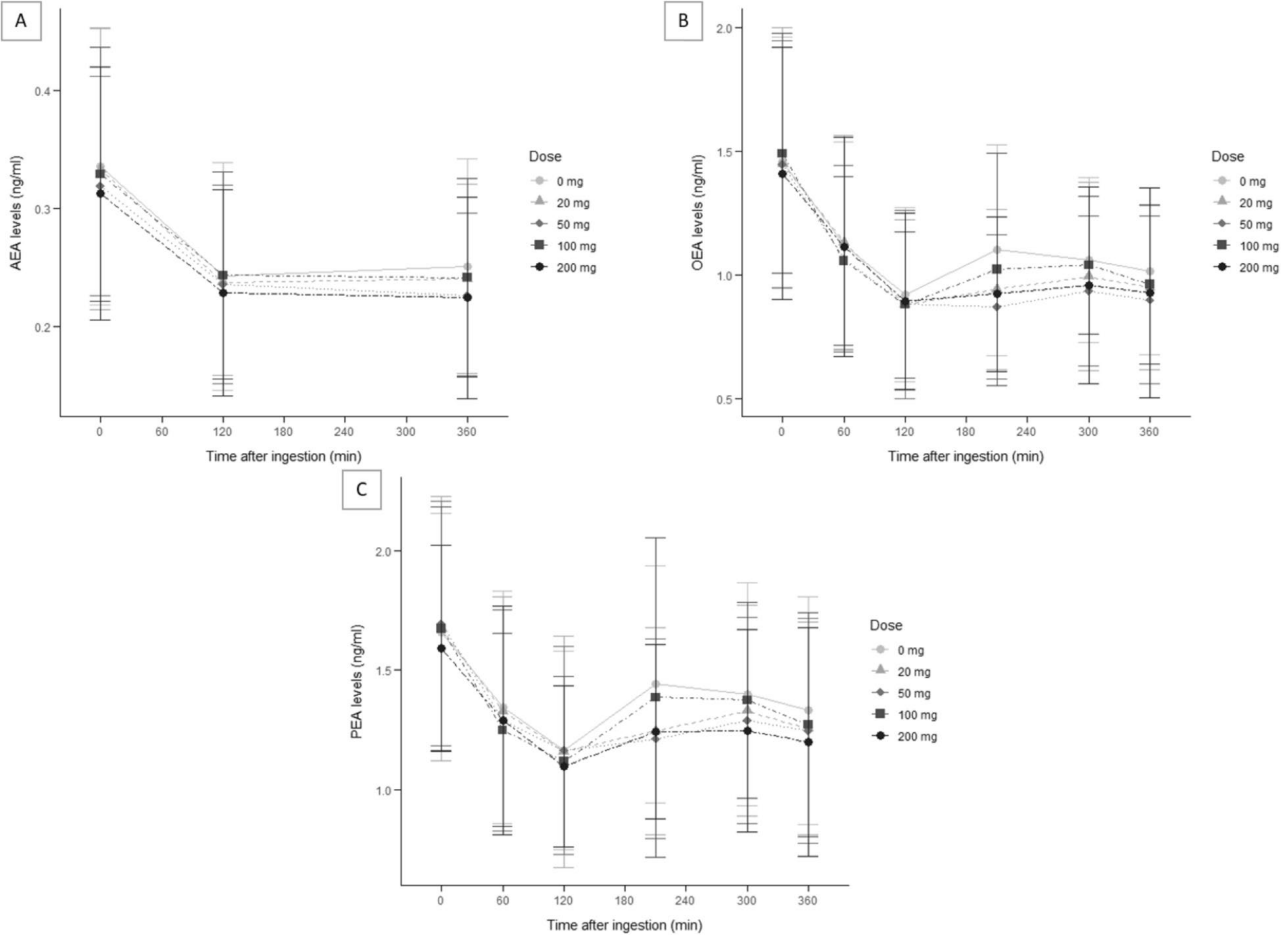
Measured NAEs levels in plasma following acute administration of 0 mg (light gray circle), 20 mg (light gray triangle), 50 mg (gray diamond), 100 mg (dark gray square) and 200 mg (black circle) of CBD oil. Mean and standard deviation (ng/ml) are presented for (A) AEA, (B) PEA, and (C) OEA through timepoint 0, 60, 120, 210, 300 and 360 min. AEA, anandamide; CBD, Cannabidiol; OEA, oleoylethanolamide; PEA, palmitoylethanolamide

Approximately 5% of time-course data was missing for AEA, PEA, and OEA due to missed visits or protocol deviations. Average plasma levels of AEA, PEA and OEA across timepoints for each CBD dose are illustrated in Fig. 1. Plasma levels for each analyte were highest at T0. Individual participant trajectories of plasma analytes across timepoints by CBD doses are also available in Supplementary Figures S1-S2.

As plasma levels fell after T0 for all three analytes, the extreme endocannabinoid level used for Model 1 was the lowest measured plasma concentration (Cmin) for each visit.

Table 2 displays the mean values and standard deviation for the Cmin and total exposure (AUCi) of each analyte across the CBD doses. AUCi was calculated based on the change from T0, resulting in only negative values.

**Table 2:** Mean and standard deviation of Cmin and AUCi for each endocannabinoid at each dose

NAE	Dose (mg)	Cmin (Mean ± SD; ng/mL)	AUCi (Mean ± SD; min*ng/mL)
AEA	0	0.212 ± 0.074	−27.0 ± 28.1
	20	0.210 ± 0.064	−28.4 ± 28.1
	50	0.202 ± 0.067	−26.1 ± 25.8
	100	0.209 ± 0.068	−26.0 ± 28.1
	200	0.192 ± 0.064	−25.3 ± 27.9
PEA	0	0.977 ± 0.314	−105 ± 125
	20	0.949 ± 0.328	−136 ± 156
	50	0.926 ± 0.292	−151 ± 135
	100	0.946 ± 0.283	−127 ± 137
	200	0.893 ± 0.262	−123 ± 114
OEA	0	0.754 ± 0.227	−135 ± 133
	20	0.708 ± 0.244	−157 ± 155
	50	0.672 ± 0.197	−172 ± 140
	100	0.719 ± 0.189	−164 ± 149
	200	0.692 ± 0.247	−149 ± 141

*AEA* Anandamide, *AUCi* Area Under the Curve to increase, *CI* Confidence Interval, *Cmin* Minimum Concentration, *NAE* N-acylethanolamine, *OEA* Oleoylethanolamide, *PEA* Palmitoylethanolamide, *SD* Standard Deviation

Table 3 displays the arithmetic mean and standard deviation of peak CBD plasma concentrations at each dose, overall and by sex. There were no significant differences between male and female participants (*p* > 0.05) across all doses. Placebo dose (0 mg) concentrations were below the limit of quantification in most participants.

**Table 3:** Mean (± SD) of peak CBD plasma concentrations across doses, overall and stratified by sex

	Dose (mg)	Cmax (overall) (Mean ± SD; ng/mL) (*N* = 70)	Cmax (females) (Mean ± SD; ng/mL) (*n* = 38)	Cmax (males) (Mean ± SD; ng/mL) (*n* = 32)
CBD	0	< 0.10	0.15 ± 0.78	< 0.10
	20	5.63 ± 4.90	4.88 ± 3.83	6.49 ± 5.84
	50	17.0 ± 14.2	16.1 ± 13.3	18.0 ± 15.4
	100	48.0 ± 36.9	46.5 ± 34.2	49.9 ± 40.3
	200	95.4 ± 89.4	101.0 ± 99.9	88.8 ± 76.2

*CBD* Cannabidiol**,** *Cmax* Maximum Concentration, *SD* Standard Deviation

### Association between Cmin and CBD doses

Model 1 examined Cmin across different CBD doses (full results reported in Supplementary Table S1). For all three NAEs, baseline levels (*p* < 0.001) and sex (*p* < 0.01) had significant effects on Cmin. *Post-hoc* analysis (Supplementary Table S2) showed no dose-dependent differences between sexes (*p* > 0.05). No significant effects were observed for dosing sequence or participant ID. Pairwise comparisons revealed no significant differences in Cmin for any analyte across doses.

### Association between AUCi and CBD doses

Model 2 investigated the AUCi of analytes across different CBD doses. There was a significant effect of CBD dose on the AUCi for both PEA and OEA (*p* < 0.05). No significant effects were observed for dosing sequence or participant ID. Further analysis of pairwise comparisons (shown in Table 4 below) revealed that, for both PEA and OEA, the placebo dose exhibited significantly higher AUCi values compared to the 50 mg CBD dose (*p* < 0.05), with a moderate effect size (PEA, Cohen’s *d* = 0.515, 95% CI [0.174; 0.857]; OEA, Cohen’s *d* = 0.586, 95% CI [0.244; 0.927]). Additionally, for both PEA and OEA, the placebo dose trended towards higher AUCi values compared to the 200 mg CBD dose (*p* < 0.100), with a small-to-moderate effect size (PEA, Cohen’s *d* = 0.432, 95% CI [0.092; 0.772]; OEA, Cohen’s *d* = 0.472, 95% CI [0.131; 0.813]). There were no significant pairwise comparisons between other doses. See Supplementary Table S3 for full Model 2 results.

**Table 4:** Pairwise comparison results for AEA, PEA and OEA AUCi estimated marginal means – transformed data from Model 2

Contrast	Cohen’s d	SE	lower.CL	upper.CL	p-value
AEA					
(0 mg—20 mg)	0.046	0.170	−0.289	0.382	0.999
(0 mg—50 mg)	0.092	0.172	−0.247	0.431	0.984
(0 mg—100 mg)	0.000	0.171	−0.337	0.337	1.000
(0 mg—200 mg)	0.180	0.172	−0.159	0.519	0.834
(20 mg—50 mg)	0.046	0.172	−0.293	0.384	0.999
(20 mg—100 mg)	−0.046	0.171	−0.383	0.291	0.999
(20 mg—200 mg)	0.134	0.172	−0.205	0.473	0.937
(50 mg—100 mg)	−0.092	0.172	−0.431	0.248	0.984
(50 mg—200 mg)	0.088	0.173	−0.253	0.429	0.987
(100 mg—200 mg)	0.180	0.173	−0.160	0.520	0.835
PEA					
(0 mg—20 mg)	0.390	0.172	0.052	0.727	0.155
(0 mg—50 mg)	0.515	0.173	0.174	0.857	0.025*
(0 mg—100 mg)	0.266	0.172	−0.072	0.604	0.529
(0 mg—200 mg)	0.472	0.173	0.131	0.813	0.051
(20 mg—50 mg)	0.126	0.172	−0.213	0.465	0.949
(20 mg—100 mg)	−0.124	0.171	−0.461	0.214	0.951
(20 mg—200 mg)	0.082	0.172	−0.257	0.421	0.989
(50 mg—100 mg)	−0.249	0.173	−0.589	0.091	0.599
(50 mg—200 mg)	−0.044	0.174	−0.385	0.298	0.999
(100 mg—200 mg)	0.206	0.173	−0.135	0.546	0.756
OEA					
(0 mg—20 mg)	0.351	0.171	0.014	0.688	0.242
(0 mg—50 mg)	0.586	0.174	0.244	0.927	0.007*
(0 mg—100 mg)	0.266	0.172	−0.072	0.604	0.529
(0 mg—200 mg)	0.432	0.173	0.092	0.772	0.091
(20 mg—50 mg)	0.234	0.172	−0.105	0.573	0.652
(20 mg—100 mg)	−0.085	0.171	−0.423	0.253	0.988
(20 mg—200 mg)	0.081	0.172	−0.258	0.419	0.990
(50 mg—100 mg)	−0.319	0.173	−0.660	0.021	0.347
(50 mg—200 mg)	−0.154	0.173	−0.495	0.187	0.901
(100 mg—200 mg)	0.166	0.173	−0.175	0.506	0.873

*AEA* Anandamide, *AUCi* Area Under the Curve to increase, *CL* Confidence Level, *Cmin* Minimum Concentration, *OEA* Oleoylethanolamide, *PEA* Palmitoylethanolamide, *SE* Standard Error

Signifies *p* < 0.100

^*^Signifies *p* < 0.050

Model 2 also reported a significant sex effect on NAEs AUCi (*p* < 0.05). *Post-hoc* subgroup analyses (Supplementary Table S4) showed dose-dependent differences between the sexes for PEA and OEA, with female participants having a significant CBD dose effect (*p* < 0.05), but not males (*p* > 0.05). Further analysis of pairwise comparisons within female subjects (shown in Table 5 below) revealed that, for both PEA and OEA, the placebo dose exhibited significantly higher AUCi values compared to the 200 mg CBD dose (*p* < 0.05), with a moderate-to-high effect size (PEA, Cohen’s *d* = 0.677, 95% CI [0.205;1.149]; OEA, Cohen’s *d* = 0.674, 95% CI [0.202; 1.145]). For 50 mg CBD, compared to placebo, the AUCi of OEA was significantly higher (*p* < 0.05) and the AUCi of PEA trended towards being higher (*p* < 0.100), with a moderate-to-high effect size for both (PEA, Cohen’s *d* = 0.601, 95% CI [0.129;1.072]; OEA, Cohen’s *d* = 0.772, 95% CI [0.299; 1.245]). *Post-hoc* subgroup analysis on AEA AUCi showed no dose-dependent differences between sexes (*p* > 0.05).

**Table 5:** Pairwise comparison results for AEA, PEA and OEA AUCi estimated marginal means within female subjects – transformed data from Model 2

Contrast	Cohen’s d	SE	lower.CL	upper.CL	*p*-value
AEA					
(0 mg—20 mg)	−0.138	0.232	−0.596	0.320	0.976
(0 mg—50 mg)	0.020	0.236	−0.445	0.486	1.000
(0 mg—100 mg)	0.011	0.235	−0.454	0.477	1.000
(0 mg—200 mg)	0.174	0.236	−0.293	0.641	0.947
(20 mg—50 mg)	0.158	0.235	−0.306	0.623	0.962
(20 mg—100 mg)	0.149	0.235	−0.315	0.614	0.969
(20 mg—200 mg)	0.312	0.237	−0.157	0.781	0.681
(50 mg—100 mg)	−0.009	0.235	−0.473	0.455	1.000
(50 mg—200 mg)	0.154	0.238	−0.318	0.625	0.967
(100 mg—200 mg)	0.163	0.237	−0.306	0.631	0.959
PEA					
(0 mg—20 mg)	0.390	0.233	−0.072	0.851	0.452
(0 mg—50 mg)	0.601	0.238	0.129	1.072	0.087
(0 mg—100 mg)	0.269	0.237	−0.200	0.738	0.786
(0 mg—200 mg)	0.677	0.239	0.205	1.149	0.038 *
(20 mg—50 mg)	0.211	0.235	−0.254	0.676	0.897
(20 mg—100 mg)	−0.121	0.235	−0.585	0.344	0.986
(20 mg—200 mg)	0.287	0.237	−0.181	0.756	0.742
(50 mg—100 mg)	−0.332	0.235	−0.797	0.134	0.621
(50 mg—200 mg)	0.076	0.238	−0.395	0.547	0.998
(100 mg—200 mg)	0.408	0.238	−0.063	0.879	0.426
OEA					
(0 mg—20 mg)	0.436	0.233	−0.025	0.897	0.334
(0 mg—50 mg)	0.772	0.239	0.299	1.245	0.012 *
(0 mg—100 mg)	0.390	0.237	−0.079	0.858	0.468
(0 mg—200 mg)	0.674	0.239	0.202	1.145	0.039 *
(20 mg—50 mg)	0.336	0.236	−0.130	0.802	0.609
(20 mg—100 mg)	−0.046	0.235	−0.510	0.419	1.000
(20 mg—200 mg)	0.238	0.237	−0.230	0.706	0.852
(50 mg—100 mg)	−0.382	0.236	−0.849	0.084	0.484
(50 mg—200 mg)	−0.098	0.238	−0.568	0.372	0.994
(100 mg—200 mg)	0.284	0.238	−0.186	0.754	0.754

*AEA* Anandamide, *AUCi* Area Under the Curve to increase, *CL* Confidence Level, *Cmin* Minimum Concentration, *OEA* Oleoylethanolamide, *PEA* Palmitoylethanolamide, *SE* Standard Error

Signifies *p* < 0.100

^*^Signifies *p* < 0.050

## Discussion

This study aimed to investigate the acute effects of CBD on plasma levels of NAEs in healthy occasional cannabis users. Specifically, we evaluated the influence of CBD at marketed low doses (20 mg to 200 mg) on AEA, PEA, and OEA. The main findings indicate that compared to placebo, CBD reduced overall exposure (AUCi) of PEA and OEA, with effects driven by female participants and, as confirmed by post-hoc subgroup analyses, evident at 50 mg and 200 mg, but showed no differences at 20 and 100 mg doses. AEA levels were not different than placebo at any of the doses tested in this study. Our data also showed that plasma levels of all three analytes decreased over time during study visits.

The lack of significant changes in AEA levels may reflect dose-dependent effects, as prior studies using higher doses of CBD (e.g., > 400 mg) have reported alterations in AEA levels (Couttas et al. 2024; Hua et al. 2023), suggesting that the doses used here (i.e., maximum 200 mg) may fall below the threshold required to significantly influence AEA metabolism. No linear dose–response relationship between CBD and plasma levels of PEA and OEA was found. Previous research has supported potential bi-phasic or multi-modal dose-dependent effects of CBD (Zuardi et al. 2017). Such responses have been documented in endocannabinoid research, where lower and higher doses of endocannabinoid modulators can produce bidirectional effects (Lafenêtre et al. 2009; Cui et al. 2015; Viveros et al. 2005). In humans, Zuardi et al. (2017) reported a U-shaped dose–response for CBD, where 300 mg reduced anxiety, while 150 mg and 600 mg had no effect compared to placebo. In the current study, both 50 mg and 200 mg doses were similarly associated with decreased PEA and OEA levels compared to placebo, though at a trend level only in some cases. It should be noted that the current study was not designed or powered for exploring the precise pharmacological mechanisms of CBD’s effects, and so these results should be interpreted with caution.

As suggested, the sex effect detected in the main model also emerged in the post-hoc subgroup analysis, with significant changes in NAE levels observed in female participants only. While preliminary, this finding is consistent with prior evidence of sex-dimorphism in the endocannabinoid system. Circulating NAE levels have been reported to differ between males and females across the lifespan, with hormonal influences such as estrogen and progesterone playing a regulatory role in endocannabinoid synthesis and degradation (Forner-Piquer et al. 2024; Craft et al. 2013; Blanton et al. 2021). In addition, sex differences in ECS-related enzymes, including FAAH, and in CB1 receptor signaling have been documented (Forner-Piquer et al. 2024; Craft et al. 2013; Rajasekera et al. 2025), which may alter CBD’s pharmacodynamics. These results suggest that sex may be an important moderator of CBD’s effects on PEA and OEA at the doses tested here. Future studies with larger, sex-stratified samples are warranted to confirm these findings and clarify the underlying biological mechanisms. Our data helps build a better understanding of the dose–response relationship of CBD in healthy subjects by supporting, in humans, what has been observed in various pre-clinical research models using different ranges of CBD doses (e.g., depression, compulsive behaviors, schizophrenia, pain, type 1 diabetes, inflammation, arthritis, pain) (Zanelati et al. 2010; Schiavon et al. 2016; Casarotto et al. 2010; Levin et al. 2025; Genaro et al. 2017; Esposito et al. 2013; Gallily et al. 2015). Considering the diverse and tissue-specific regulatory roles of the endocannabinoid system, our results contextualized with pre-clinical data support the notion that from different physiological contexts emerges different dose–response curves depending on the tissue, the phenotype of interest and the specific underlying biological mechanisms at play. Future crossover studies designed to capture detailed time-course changes in endogenous levels across a wider range of CBD doses (e.g., 10 mg—600 mg) could help clarify the dose–response relationship between plasma NAE levels and CBD consumption in healthy people. This approach will offer the possibility of identifying specific dose ranges necessary to generate measurable changes in endocannabinoidome-related compounds and to establish if and how changes in plasma CBD levels can be robustly associated with specific changes in neurobiological phenotypes.

The observed reductions in all NAE levels following CBD administration could be due to multiple overlapping mechanisms. First, the effect of food intake may have mediated the fall in plasma concentrations after T0. The participants consumed a standardized high-fat meal immediately before CBD ingestion in order to maximize its bioavailability (Saals et al. 2025). However, this standardized meal also presents a limitation: the ingestion of a high-fat meal can itself alter plasma levels of NAEs, independently of CBD administration. Previous studies have shown that consuming a meal, particularly one rich in lipids, can induce changes in circulating endocannabinoid levels due to an increase in circulating free fatty acids (Díaz-Rúa et al. 2020; Kuipers et al. 2019). Consequently, the majority of the variations observed in plasma concentrations of NAEs are likely attributable to postprandial effects rather than to drug administration. Additionally, although the crossover study design was chosen to reduce inter-individual variability, we did not document whether the meal was fully consumed prior to study drug ingestion. This information could have allowed for better control of this potential confounding factor.

The overall decline observed in AEA, PEA, and OEA plasma levels in all experimental conditions, including placebo, might be due to the functional links between NAEs and the gut-brain-axis, that are governed by intricate regulatory networks tightly linked with daily and seasonal rhythmical cycles (Sharkey and Wiley 2016; Marzo 2020; Vaughn et al. 2010). Our results differ from prior findings showing these analytes typically rise from morning to mid-afternoon before returning to baseline-like levels (Hillard 2018). Specifically, Hanlon (2020) reported that natural AEA levels were lowest around 10 AM (around 0.65 ng/ml) and rose until 3 PM (around 0.9 ng/ml), suggesting a robust circadian influence. However, as our study visits began at 9 AM with a similar AEA basal level, this pattern does not explain the observed changes from T0. This could possibly be explained by the required 12-h fasting period before visits, which may have elevated baseline endocannabinoid levels, followed by a decline after food intake (Hanlon 2020; Silvestri and Di Marzo 2013). It could also be explained by the fact that Hanlon et al. gave their participants a high-carbohydrate meal, whereas participants in the present study were provided with a high-fat meal, which would have a larger influence on NAE levels (Matias et al. 2008; Starowicz et al. 2008).

In terms of the comparative decrease in plasma PEA and OEA levels compared to placebo observed in our data, one possible explanation may be interactions between CBD and the transient receptor potential vanilloid type 1 (TRPV1). Previous studies suggest that direct TRPV1 activation can lead to a time- and dose-dependent reduction in NAE synthesis (Manchanda et al. 2021). At the low doses tested in this study, CBD may act as a partial TRPV1 activator rather than a full agonist, like chemicals such as capsaicin, resulting in a moderate but sustained reduction in these signaling lipids (Starkus et al. 2019; Simon et al. 2005; Louis-Gray et al. 2022). Additionally, CBD’s ability to modulate membrane fluidity or intracellular signaling may contribute to TRPV1 stabilization, possibly altering downstream effects on NAEs (Ghovanloo et al. 2021; Mayar et al. 2022; Maccarrone et al. 2011). However, this possible mechanism remains speculative, as no data on TRPV1 activation or modulation were collected in this study, and no linear dose–effect of CBD on NAEs was found. In addition, TRPV1 modulation alone could not fully account for the sustained decreases in circulating levels of these mediators observed across multiple doses, which supports the notion that the many regulatory roles played by the endocannabinoid system are accomplished by a vast array of endogenous bioactive compounds that act synergistically (Ben-Shabat et al. 1998), highlighting the need to develop more integrated analytical approaches that take into consideration coordinated physiological changes affecting hundreds of endocannabinoid-related mediators simultaneously.

A second putative mechanism that could partially explain our results is the potential role of FABPs in modulating endocannabinoid levels following CBD administration. Specifically, CBD may inhibit FABPs from transporting AEA, PEA, and OEA to their catabolic enzyme, FAAH, thereby increasing their availability (Elmes et al. 2015; Kaczocha et al. 2012). While this mechanism could account for the observed increase in AEA, PEA, and OEA concentrations after the T120 timepoints shown in Fig. 1, it does not fully explain the initial decrease observed immediately following CBD ingestion, which is more plausibly related to the effect of the high-fat meal that was ingested with each CBD dose. Alternatively, the V-shaped pattern observed (from an initial decrease to a subsequent increase after T120) may simply reflect a return to baseline or a natural temporal fluctuation in NAE levels rather than a pharmacological effect of CBD per se. This interpretation is supported by the fact that a similar pattern was also observed in the placebo condition, suggesting that factors unrelated to CBD administration, such as metabolic or circadian influences, could underlie this trajectory.

Our findings could also differ from studies that report increases in endocannabinoid levels following CBD administration due to differences in study design, populations, and dosing. For instance, studies on chronic cannabis users or patients with specific conditions (e.g., anxiety, schizophrenia) might observe different outcomes due to altered baseline endocannabinoid tone and consequential changes in endocannabinoid-related regulatory patterns (Bluett et al. 2014; Dlugos et al. 2012; Potvin et al. 2020; Minichino et al. 2019). Additionally, other studies often used higher doses of CBD or co-administered THC (Chester et al. 2024; Viveros et al. n.d.; De Petrocellis et al. 2011), which more directly influences endocannabinoid levels via CB1 receptor affinity and competitive interaction with AEA (Martin-Willett et al. 2024; Watkins 2019). Effects of low CBD doses on plasma NAE levels represented crucial missing information in the broader understanding of pharmacological interactions between exogenous and endogenous cannabinoids. Our results can be compared to previous findings using higher CBD doses, offering novel additional evidence highlighting the importance of interactions between dose and population-specific factors.

### Limitations

Several limitations should be acknowledged. First, 2-arachidonoylglycerol (2-AG), a principal endocannabinoid alongside AEA, was not measured in this study due to budget limitations, which could have provided a more comprehensive profile of endocannabinoid system activity (Baggelaar et al. 2018). Second, the number of timepoints was limited, particularly for AEA levels, which may have reduced sensitivity to detect fluctuations. Based on Fig. 1, PEA and OEA levels seem to show possible differences between CBD doses at timepoints T210 and T300. Samples at these timepoints were not analyzed for AEA, potentially indicating a missed effect.

Third, NAEs are labile at room temperature and may undergo ex vivo degradation (Vogeser et al. 2006; Pasella et al. 2013). It is therefore recommended to immediately cool samples and perform centrifugation at 4 °C to preserve their initial concentrations (Fanelli et al. 2012). Literature suggests that sequential plasma sampling from incubated blood can detect dynamic changes in AEA between 30 and 120 min after venipuncture (Pruessner et al. 2003). Our samples were processed within 20 min of drawing, and this likely minimized ex vivo changes. While our procedure may have, in theory, impacted AEA quantification, all samples were treated consistently, allowing valid relative comparisons between CBD doses. Baseline concentrations were similar to those reported in other studies, suggesting our measurements fall within expected ranges (Fanelli et al. 2012; Quantification of 24 circulating endocannabinoids, endocannabinoid-related compounds, and their phospholipid precursors in human plasma by UHPLC-MS, MS. 2019; Ottria et al. 2014).

It should also be noted that plasma endocannabinoid levels may not fully correlate with concentrations in the brain, where these molecules are produced and utilized locally for tissue-specific functions (De Petrocellis et al. 2011; Watkins 2019). Additionally, caffeine use and whether participants finished the study meal in its entirety was not accounted for in the statistical analysis, which could represent a potential confounding factor (Díaz-Rúa et al. 2020; Kuipers et al. 2019; Rossi et al. 2009).

Individual differences, such as nicotine use (Cippitelli et al. 2011), body fat percentage (Chayasirisobhon 2021), genetics (Pratt-Hyatt et al. 2010; Diao et al. 2022) and menstrual cycle (Kirschbaum et al. 1999), were not assessed in this study, despite their known impact on endocannabinoid signaling. In addition, we observed large inter-personal heterogeneity in NAE fluctuations across visits, indicating subgroup differences that were not further analyzed except for sex; detailed patterns are provided in Supplementary Figures S1–S3.

Finally, although this study detected statistically significant changes in circulating PEA and OEA levels through time following CBD ingestion, the functional biological significance of the changes remains to be determined. The magnitude of change was modest, and no behavioral or physiological outcomes were used to assess the potential functional consequences. As such, we cannot infer whether the observed plasma level changes translate into measurable effects on stress regulation, mood or other psychosocial outcomes.

## Conclusion

To our knowledge, this is the first study to evaluate the acute effects of low-dose CBD on circulating NAE levels in healthy, occasional cannabis users. Our findings reveal a sex-dependant differential effect of CBD on PEA and OEA. Compared to placebo, CBD significantly reduced overall exposure to PEA and OEA, but not AEA, an effect detected only in females at 50 mg and 200 mg. These results contribute to a growing understanding of CBD’s interactions with the endocannabinoid system, particularly at doses reflective of occasional cannabis use. They also highlight the complex nature of CBD’s pharmacological effects, influenced by dose, sex and contextual factors such as stress or fasting. Future research should explore these effects in diverse populations, including individuals with altered endocannabinoid tone or metabolic conditions, while also examining the role of dietary and stress-related factors in modulating endocannabinoid responses to CBD, and whether changes in endocannabinoid levels are linked to behavioural or physiological assessments. Such integrated approaches would help clarify the clinical relevance of the observed biochemical effects.

## Supplementary Information

Supplementary Material 1.

## Data Availability

The datasets used and analysed during the current study are available from the corresponding author on reasonable request.
