# Supplementary material for: Sex-specific association between low oral doses of cannabidiol (CBD) and plasma concentration of anandamide (AEA), N-palmitoylethanolamine (PEA) and N-oleoylethanolamine (OEA) in healthy occasional cannabis users
Source: J Cannabis Res. 2026 Jan 9;8:20. doi: 10.1186/s42238-025-00356-x (PMC12879445; doi:10.1186/s42238-025-00356-x)

Online Supplementary Material

Title: Sex-Specific association between low oral doses of cannabidiol (CBD) and plasma concentration of anandamide (AEA), N-palmitoylethanolamine (PEA) and N-oleoylethanolamine (OEA) in healthy occasional cannabis users

Anita Abboud[1,2], Lucy Chester[1,2], Francois-Olivier Hébert[2], and Didier Jutras-Aswad*[1,2]

[1]Department of Psychiatry and Addictology, Université de Montréal, Montréal, Québec, Canada;

[2]Research Centre, Centre hospitalier de l'Université de Montréal, Montréal, Québec, Canada;

Corresponding author:
Didier Jutras-Aswad
didier.jutras-aswad@umontreal.ca

Table of contents

Supplementary Table S1: Results from GLMM model 1 3

Supplementary Table S2: *Post-hoc* exploratory subgroup analysis by sex using Model 1. 5

Supplementary Table S3: Results from GLMM model 2 7

Supplementary Table S4: *Post-hoc* exploratory subgroup analysis by sex using Model 2. 9

Supplementary Figure S1. Individual Trajectories of Plasma AEA Levels 11

Supplementary Figure S2. Individual Trajectories of Plasma PEA Levels 12

Supplementary Figure S3. Individual Trajectories of Plasma OEA Levels 13

Supplementary Method S1: Analytical Methodology for AEA 14

Supplementary Method S2: Analytical Methodology for PEA and OEA 20

Supplementary Table S1: Results from GLMM model 1 showing the association between Cmin and CBD doses for each analyte.

**AEA**					
		**Estimate**	**SE**	**t value**	**p-value**
**(Intercept)**		0.099	0.015	6.750	
**Dose**					0.501
	**20 mg**	-0.001	0.009	-0.119	
	**50 mg**	-0.004	0.009	-0.451	
	**100 mg**	-0.002	0.009	-0.225	
	**200 mg**	-0.014	0.009	-1.603	
**Visit**					**0.000** ***
	**V2**	0.029	0.009	3.258	
	**V3**	0.029	0.009	3.261	
	**V4**	0.041	0.009	4.573	
	**V5**	0.026	0.009	2.895	
**Sex**		-0.036	0.007	-4.848	**0.000** ***
**T0**		0.330	0.030	10.947	**< 2.2e-16** ***
**PEA**					
**(Intercept)**		0.616	0.065	9.470	
**Dose**					0.302
	**20 mg**	-0.036	0.034	-1.066	
	**50 mg**	-0.058	0.034	-1.709	
	**100 mg**	-0.040	0.034	-1.169	
	**200 mg**	-0.070	0.034	-2.037	
**Visit**					0.458
	**V2**	0.028	0.035	0.813	
	**V3**	0.027	0.035	0.787	
	**V4**	0.054	0.035	1.552	
	**V5**	-0.003	0.034	-0.084	
**Sex**		-0.151	0.040	-3.761	**0.000** ***
**T0**		0.258	0.028	9.330	**<2e-16** ***
**OEA**					
**(Intercept)**		0.497	0.051	9.763	
**Dose**					0.110
	**20 mg**	-0.048	0.031	-1.570	
	**50 mg**	-0.082	0.031	-2.664	
	**100 mg**	-0.045	0.031	-1.476	
	**200 mg**	-0.058	0.031	-1.879	
**Visit**					0.382
	**V2**	0.013	0.031	0.425	
	**V3**	0.029	0.031	0.920	
	**V4**	0.058	0.032	1.825	
	**V5**	0.009	0.031	0.276	

**Sex**		-0.081	0.029	-2.765	**0.006 ***
**T0**		0.196	0.024	8.290	**3.481e-15 *****
**AEA, anandamide; Cmin, Minimum Concentration; OEA, oleoylethanolamide; PEA, palmitoylethanolamide; SE, Standard Error; T0, Pre-ingestion timepoint * Signifies p < 0.050; *** Signifies p < 0.001**					

Visit effect: A significant visit effect was observed for AEA Cmin ($p < 0.001$), with post-hoc analyses showing this effect specifically for visit 4 compared to visit 1 (Cohen's d = -0.7835, 95% CI [-1.1321; -0.435]).

Supplementary Table S2: *Post-hoc* exploratory subgroup analysis by sex using Model 1.

		Males				Females			
**AEA**									
		**Estimate**	**SE**	**t value**	**p-value**	**Estimate**	**SE**	**t value**	**p-value**
**(Intercept)**		0.088	0.024	3.748		0.070	0.018	3.884	
**Dose**					0.693				0.502
	**20 mg**	-0.013	0.014	-0.934		0.010	0.012	0.833	
	**50 mg**	-0.011	0.014	-0.782		0.004	0.012	0.318	
	**100 mg**	-0.008	0.014	-0.591		0.003	0.012	0.259	
	**200 mg**	-0.020	0.014	-1.450		-0.011	0.012	-0.947	
**Visit**					0.066 .				**0.009 ***
	**V2**	0.034	0.014	2.417		0.023	0.012	1.953	
	**V3**	0.028	0.014	2.039		0.029	0.012	2.401	
	**V4**	0.037	0.014	2.650		0.044	0.012	3.613	
	**V5**	0.023	0.014	1.657		0.024	0.012	2.027	
**T0**		0.383	0.053	7.290	**3.097e-13 *****	0.295	0.037	7.885	**3.135e-15 *****
**PEA**									
**(Intercept)**		0.559	0.093	5.990		0.489	0.085	5.741	
**Dose**					0.809				0.211
	**20 mg**	-0.033	0.049	-0.677		-0.031	0.046	-0.662	
	**50 mg**	-0.027	0.051	-0.522		-0.064	0.047	-1.359	
	**100 mg**	-0.062	0.051	-1.231		-0.023	0.047	-0.489	
	**200 mg**	-0.040	0.050	-0.802		-0.104	0.047	-2.199	
**Visit**					0.504				0.087 .
	**V2**	0.004	0.051	0.081		0.052	0.047	1.115	
	**V3**	-0.073	0.050	-1.481		0.101	0.049	2.076	
	**V4**	-0.002	0.051	-0.031		0.101	0.048	2.101	
	**V5**	-0.025	0.050	-0.503		0.008	0.048	0.171	
**T0**		0.312	0.043	7.233	**4.733e-13 *****	0.228	0.037	6.209	**5.334e-10 *****
**OEA**									

**(Intercept)**		0.461	0.074	6.232		0.421	0.070	6.029	
**Dose**					0.901				0.085 .
	**20 mg**	-0.044	0.046	-0.947		-0.047	0.041	-1.138	
	**50 mg**	-0.038	0.048	-0.788		-0.108	0.042	-2.583	
	**100 mg**	-0.034	0.047	-0.710		-0.062	0.042	-1.468	
	**200 mg**	-0.026	0.047	-0.547		-0.094	0.042	-2.235	
**Visit**					0.721				0.175
	**V2**	-0.003	0.048	-0.071		0.038	0.042	0.897	
	**V3**	-0.041	0.046	-0.890		0.083	0.044	1.906	
	**V4**	0.026	0.048	0.549		0.095	0.044	2.162	
	**V5**	-0.008	0.047	-0.174		0.031	0.043	0.733	
**T0**		0.228	0.038	6.041	**1.534e-09 *****	0.184	0.031	5.899	**3.656e-09 *****

AEA, anandamide; Cmin, Minimum Concentration; OEA, oleoylethanolamide; PEA, palmitoylethanolamide; SE, Standard Error; T0, Pre-ingestion timepoint
. Signifies p < 0.100; * Signifies p < 0.050; * Signifies p < 0.001**

Supplementary Table S3: Results from GLMM model 2 showing the association between AUCi and CBD doses for each analyte.

**AEA**					
		**Estimate**	**SE**	**t value**	**p-value**
**(Intercept)**		37.162	4.711	7.888	
**Dose**					0.810
	**20 mg**	-2.096	2.816	-0.744	
	**50 mg**	-2.062	2.845	-0.725	
	**100 mg**	-0.439	2.828	-0.155	
	**200 mg**	-3.046	2.845	-1.071	
**Visit**					0.014 *
	**V2**	6.675	2.879	2.319	
	**V3**	5.077	2.859	1.776	
	**V4**	9.975	2.873	3.472	
	**V5**	4.972	2.827	1.759	
**Sex**		-11.813	2.402	-4.918	**8.76e-07 *****
**T0**		-187.273	9.675	-19.356	**< 2e-16 *****
**PEA**					
**(Intercept)**		281.659	24.3	11.591	
**Dose**					**0.019 ***
	**20 mg**	-29.417	12.485	-2.356	
	**50 mg**	-37.859	12.588	-3.008	
	**100 mg**	-19.906	12.523	-1.59	
	**200 mg**	-34.848	12.584	-2.769	
**Visit**					0.854
	**V2**	13.386	12.738	1.051	
	**V3**	6.295	12.73	0.495	
	**V4**	11.507	12.8	0.899	
	**V5**	6.291	12.577	0.5	
**Sex**		-45.755	15.478	-2.956	**0.003 ***
**T0**		-221.857	10.299	-21.542	**< 2e-16 *****
**OEA**					
**(Intercept)**		216.780	19.103	11.348	
**Dose**					**0.014 ***
	**20 mg**	-23.890	11.073	-2.158	
	**50 mg**	-38.653	11.163	-3.463	
	**100 mg**	-20.756	11.116	-1.867	
	**200 mg**	-25.614	11.155	-2.296	
**Visit**					0.511
	**V2**	8.088	11.409	0.709	
	**V3**	16.192	11.312	1.431	
	**V4**	18.641	11.525	1.617	
	**V5**	11.630	11.211	1.037	

**Sex**		-29.958	11.812	-2.536	**0.011 ***
**T0**		-237.486	8.821	-26.922	**< 2e-16 *****
**AEA, anandamide; AUCi, Area Under the Curve to increase; OEA, oleoylethanolamide; PEA, palmitoylethanolamide; SE, Standard Error; T0, Pre-ingestion timepoint . Signifies p < 0.100; * Signifies p < 0.050; *** Signifies p < 0.001**					

Visit effect: A significant visit effect was observed for AEA AUCi ($p < 0.001$), with post-hoc analyses showing this effect specifically for visit 4 compared to visit 1 (Cohen's *d* = -0.585, 95% CI [-0.9318, -0.2389]).

Supplementary Table S4: *Post-hoc* exploratory subgroup analysis by sex using Model 2.

		Males				Females			
**AEA**									
		**Estimate**	**SE**	**t value**	**p-value**	**Estimate**	**SE**	**t value**	**p-value**
**(Intercept)**		31.530	7.234	4.358		28.323	6.029	4.697	
**Dose**					0.575				0.917
	**20 mg**	-4.292	4.140	-1.037		0.151	3.918	0.039	
	**50 mg**	-2.240	4.355	-0.514		-1.108	3.983	-0.278	
	**100 mg**	2.021	4.251	0.475		-1.959	3.981	-0.492	
	**200 mg**	-2.907	4.179	-0.696		-3.169	3.992	-0.794	
**Visit**					0.082 .				0.228
	**V2**	9.346	4.272	2.188		4.786	4.024	1.189	
	**V3**	6.037	4.159	1.452		4.559	4.056	1.124	
	**V4**	11.512	4.248	2.710		9.591	4.059	2.363	
	**V5**	6.324	4.230	1.495		4.201	4.020	1.045	
**T0**		-173.897	16.155	-10.765	**< 2e-16 *****	-195.124	12.463	-15.657	**<2e-16 *****
**PEA**									
**(Intercept)**		256.967	32.298	7.956		247.416	33.558	7.373	
**Dose**					0.586				**0.050 .**
	**20 mg**	-24.797	16.551	-1.498		-30.848	18.408	-1.676	
	**50 mg**	-21.037	17.282	-1.217		-44.417	18.717	-2.373	
	**100 mg**	-22.566	16.982	-1.329		-15.463	18.721	-0.826	
	**200 mg**	-19.515	16.700	-1.169		-48.517	18.689	-2.596	
**Visit**					0.502				0.652
	**V2**	8.931	17.304	0.516		19.378	18.667	1.038	
	**V3**	-17.737	16.661	-1.065		23.262	19.248	1.209	
	**V4**	4.195	17.187	0.244		18.715	19.106	0.980	
	**V5**	9.207	16.954	0.543		3.184	18.945	0.168	
**T0**		-206.866	14.973	-13.816	**<2e-16 *****	-229.778	14.470	-15.880	**< 2e-16 *****
**OEA**									
**(Intercept)**		187.325	25.163	7.445		200.050	27.570	7.257	

**Dose**					0.724				**0.025 ***
	**20 mg**	-12.610	15.044	-0.838		-31.310	16.100	-1.944	
	**50 mg**	-21.349	15.707	-1.359		-49.550	16.360	-3.028	
	**100 mg**	-15.261	15.428	-0.989		-27.930	16.370	-1.706	
	**200 mg**	-8.340	15.195	-0.549		-43.880	16.360	-2.682	
**Visit**					0.779				0.315
	**V2**	5.594	15.713	0.356		14.530	16.590	0.876	
	**V3**	-8.621	15.102	-0.571		34.210	17.060	2.005	
	**V4**	11.356	15.560	0.730		28.520	17.160	1.662	
	**V5**	3.129	15.398	0.203		20.300	16.740	1.213	
**T0**		-217.183	12.966	-16.751	**<2e-16 *****	-246.030	12.340	-19.940	**< 2e-16 *****

AEA, anandamide; Cmin, Minimum Concentration; OEA, oleoylethanolamide; PEA, palmitoylethanolamide; SE, Standard Error; T0, Pre-ingestion timepoint
. Signifies p < 0.100; * Signifies p < 0.050; * Signifies p < 0.001**

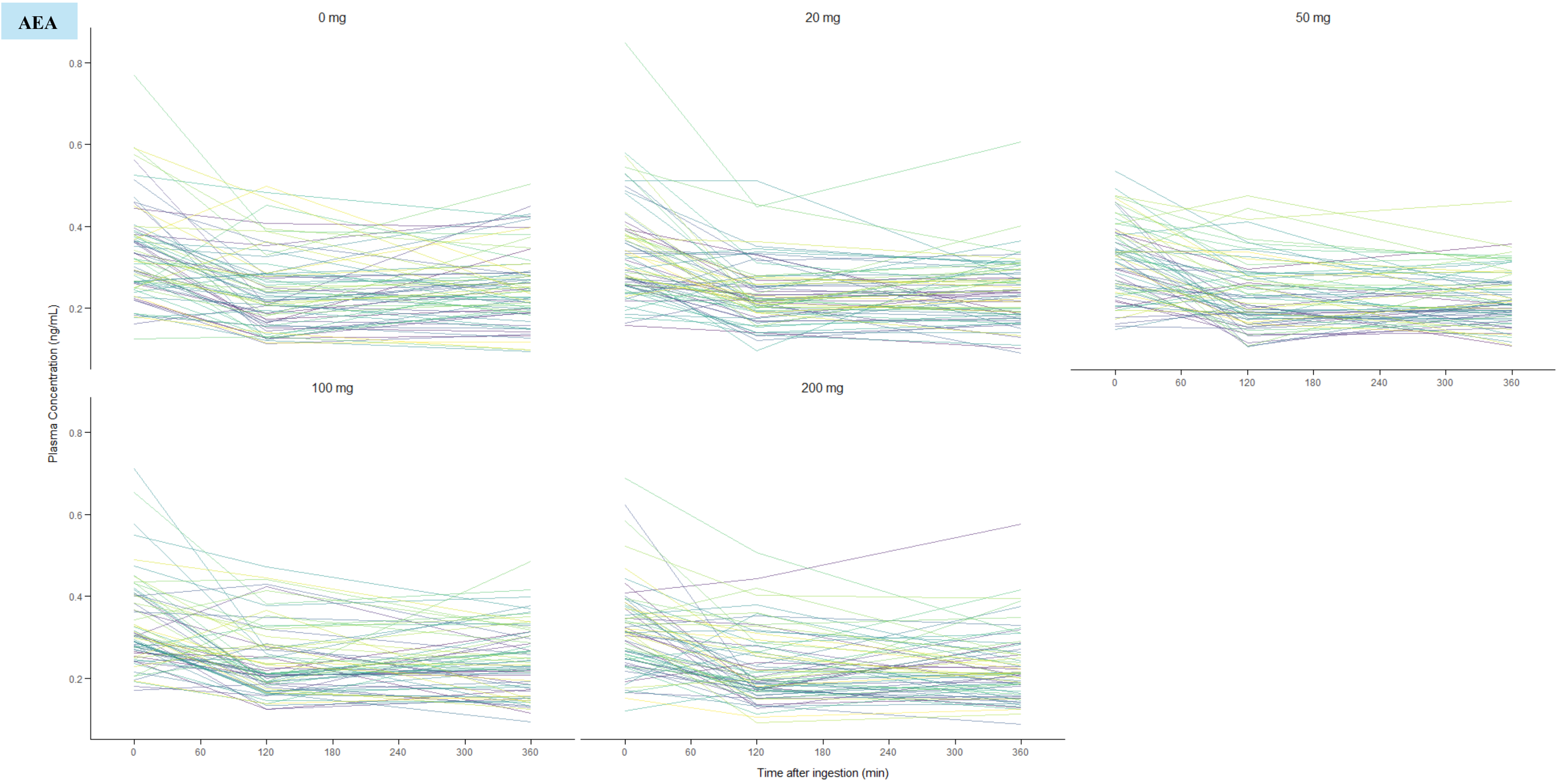

Supplementary Figure S1. Individual Trajectories of Plasma AEA Levels Across Timepoints by CBD Dose.

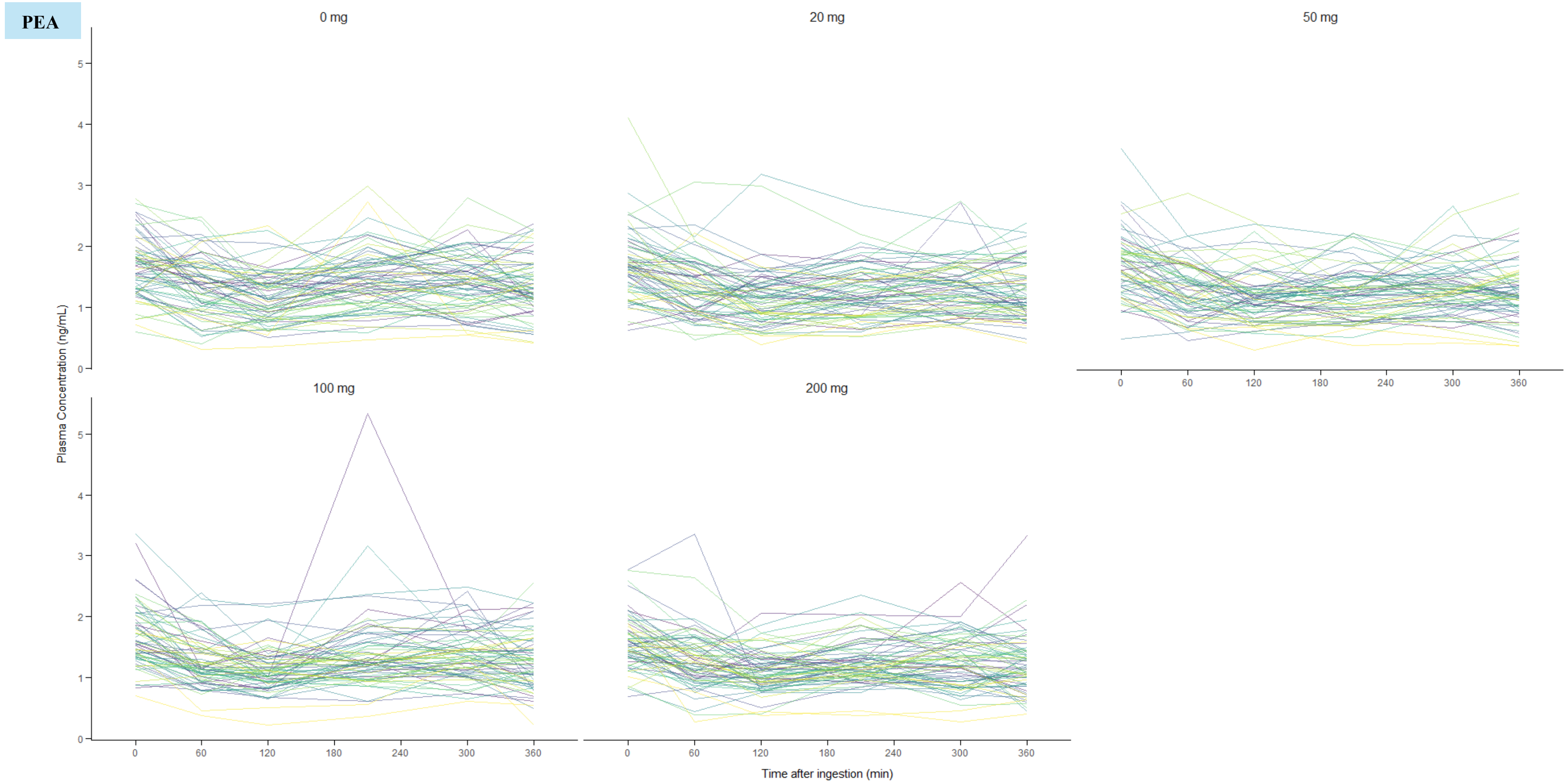

Supplementary Figure S2. Individual Trajectories of Plasma PEA Levels Across Timepoints by CBD Dose.

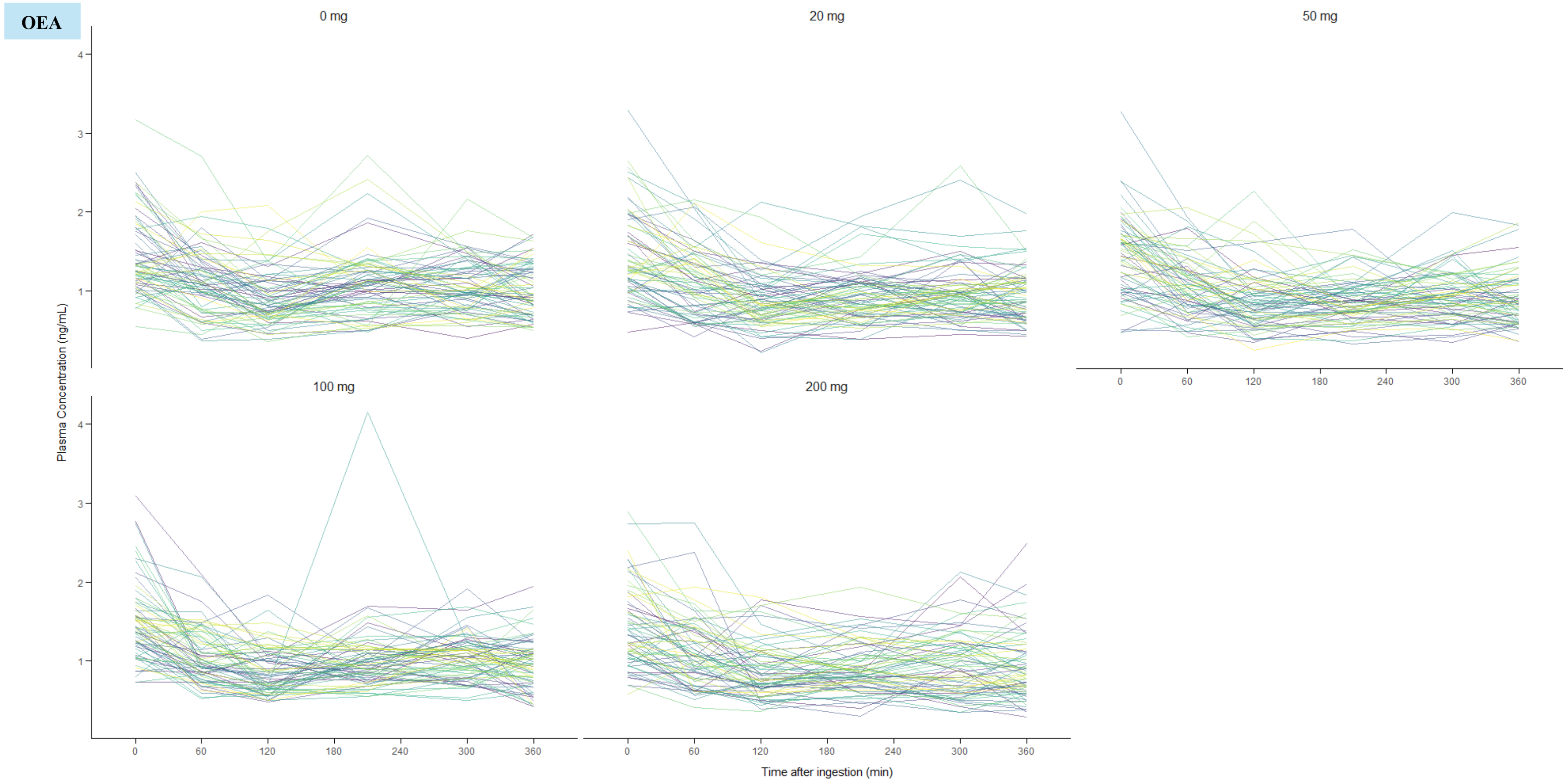

Supplementary Figure S3. Individual Trajectories of Plasma OEA Levels Across Timepoints by CBD Dose.

Supplementary Method S1: Analytical Methodology for AEA

Title: **LC-MS/MS Method for the determination of Arachidonoyl ethanolamide in Human Plasma.**

Project: 2023-10502_CBD-LD_ING

François-Olivier Hébert Ph.D.
Research Associate
Faculty of Medecine
Departement of Psychiatry and Addiction
Université de Montréal

Didier Jutras-Aswad M.D., M.Sc.
Researcher-clinician Associate Professor
Faculty of Medecine
Departement of Psychiatry and Addiction
Université de Montréal

Author:

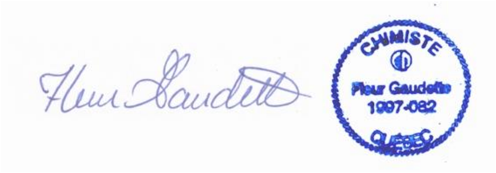

Fleur Gaudette, M.Sc. Chemist
Supervisor
Pharmacokinetics Core Facility
CRCHUM

Date: 2024-11-26

TABLE OF CONTENTS

1. Chemical Composition 16
2. Analytical Procedure 17
   2.1 Reagents 17
   2.2 Sample preparation 17
   2.3 Chromatographic conditions 17
   2.4 Mass spectrometric conditions 18
3. Chromatograms 19

LIST OF FIGURES

Figure 1: Chemical structures, formulae, and molecular weights of d_0-AEA, d_4-AEA and d_8-AEA. 16
Figure 2: Reconstructed ion chromatograms for d_4-AEA m/z 352.3 → 66.1, d_8-AEA (IS) 356.4 → 63.1 and d_0-AEA 348.3 → 62.1 19

LIST OF TABLES

Table 1: Mass spectrometry operating conditions 18

Arachidonoyl ethanolamide in Human Plasma
(*Dr. Didier Jutras-Aswad*)

Chemical Composition

The chemical structures, formulae, and molecular weights of arachidonoyl ethanolamide (d_0-AEA) and it's isotopologues d_4-AEA and d_8-AEA are depicted in figure 1.

Figure 1: **<u>Chemical structures, formulae, and molecular weights of d_0-AEA, d_4-AEA and d_8-AEA.</u>**

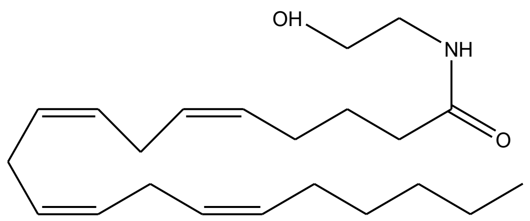

d_0-AEA (target analyte)
$C_{22}H_{37}NO_2$
Mol. Wt.: 347.53

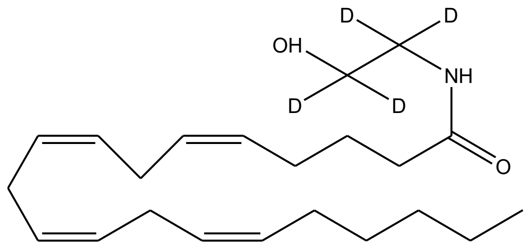

d_4-AEA (surrogate analyte)
$C_{22}H_{33}D_4NO_2$
Mol. Wt.: 351.56

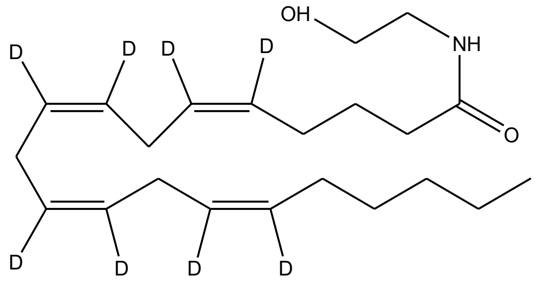

d_8-AEA (internal standard)
$C_{22}H_{29}D_8NO_2$
Mol. Wt.: 355.58

Analytical Procedure

Quantitation of biomarkers by LC–MS/MS is complicated by the presence of endogenous analytes in the matrix being analyzed. Due to the endogenous nature of d_0-AEA, a surrogate analyte approach will be used. This approach involves the use of two stable-isotope-labeled standards (d_4-AEA and d_8-AEA) to be used as a surrogate analyte and internal standard enabling calibration in the actual biological matrix.

Reagents

d_0-AEA, d_4-AEA and d_8-AEA were purchased from Cayman Chemical (Ann Arbor, MI, USA) and received in ampoules as certified solutions containing 50.0 mg/mL, 5.0 mg/mL and 1.0 mg/mL dissolved in ethanol for d_0-AEA, d_4-AEA and in methyl acetate for d_8-AEA. Human plasma containing EDTA as anticoagulant was purchased from Bioreclamation (Westbury, NY, USA). Other chemicals, including, methanol, acetonitrile, ammonium formate and water were purchased from Fisher Scientific (Fair Lawn, NJ, USA).

Sample preparation

Using a protein precipitation as sample preparation technique, d_0-AEA was extracted from human plasma. One thousand microliters of internal standard solution (2.4 ng/mL d_8-AEA in methanol) was added to an aliquot of two hundred and fifty microliters of sample. The sample was vortexed for approximately 5 seconds and let stand for a period of 10 minutes, then centrifuged at 16000 *g* for 10 minutes. The supernatant was transferred into a clean 16 x 100 mm borosilicate tube and evaporated to dryness at 40°C under a gentle stream of nitrogen. The dried extract was resuspended with 80 μL of 50% (v/v) methanol in water solution and transferred to an injection vial for analysis.

Chromatographic conditions

A gradient mobile phase was used with an Agilent Zorbax Eclipse Plus C18 RRHD analytical column (100 x 2.1 mm I.D., 1.8 μm) operating at 40°C. The initial mobile phase condition consisted of acetonitrile containing 0.1% (v/v) formic acid and 10 mM of ammonium formate in type 1 water pH 3.0 at a ratio of 60:40, respectively, and this ratio was maintained for 1 min. From 1 to 5 min a linear gradient was applied up to a ratio of 95:5 and maintained for 1 min. At 6.1 min, the mobile phase composition was reverted to the original conditions and the column was allowed

to equilibrate for 2 min for a total run time of 8 min. The flow rate was fixed at 300 µL/min and d_0-AEA and its isotopically labelled standards (d_4-AEA and d_8-AEA) eluted at 5.3 min. Five microliters of the extracted sample was injected and the total run time was set at 12 min.

Mass spectrometric conditions

The mass spectrometer was interfaced with the UPLC system using a pneumatic assisted heated electrospray ion source. MS detection was performed in positive ion mode, using selected reaction monitoring (SRM). In order to optimize the MS/MS parameters, standard solutions of d_0-AEA, d_4-AEA and d_8-AEA were infused into the mass spectrometer. The following parameters were obtained. Nitrogen was used for the sheath and auxiliary gases and was set at 50 and 15 arbitrary units. The HESI electrode was set to 3500V. The capillary and vaporizer temperatures were set at 350°C and 400°C, respectively. Argon was used as collision gas at a pressure of 2.5 mTorr. The precursor-ion reactions and the collision energy for d_0-AEA, d_4-AEA and d_8-AEA are in table 1. Total cycle time was set at 0.25 seconds. Peak width of Q1 and Q3 were both set at 0.7 FWHM.

Table 1: Mass spectrometry operating conditions

Compound	Precursor (m/z)	Product (m/z)	Collision Energy (V)	RF Lens (V)
d_0-AEA	348.3	62.1	15	64
d_4-AEA	352.3	66.1	15	65
d_8-AEA	356.3	63.1	15	66

Chromatograms

Representative chromatograms obtained upon analysis of blank human plasma, a zero standard and an extracted LLOQ standard are shown in figure 2. The overlay of an extracted LLOQ standard (blue line) and an extracted blank human plasma sample (red line) for the SRM transition of d_4-AEA are shown in figure 2a. An overlay of an extracted zero standard (blue line) and an extracted blank human plasma sample (red line) for the SRM transition of d_8-AEA are in figure 2b and the endogenous level of d_0-AEA in the blank human plasma lot extracted are shown in figure 2c.

Figure 2: **Reconstructed ion chromatograms for d_4-AEA m/z 352.3 → 66.1, d_8-AEA (IS) 356.4 → 63.1 and d_0-AEA 348.3 → 62.1**

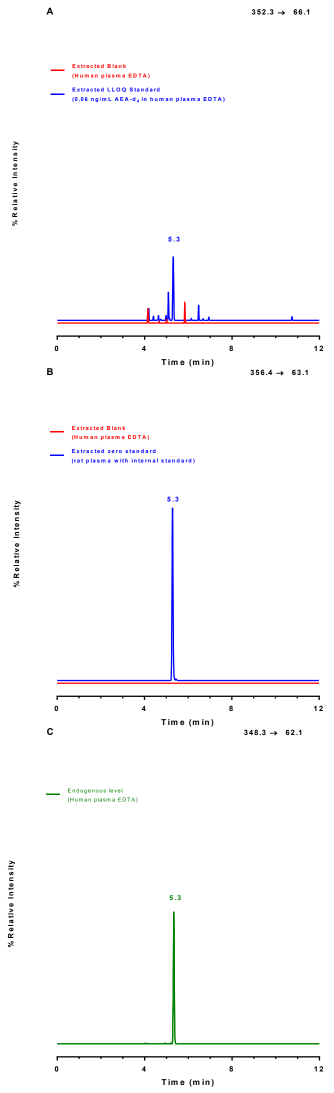

Supplementary Method S2: Analytical Methodology for PEA and OEA

Title: **LC-MS/MS Method for the determination of Palmitoyl Ethanolamide and Oleoyl Ethanolamide in Human Plasma.**

Project: 2023-10502_CBD-LD_ING

François-Olivier Hébert Ph.D.
Research Associate
Faculty of Medecine
Departement of Psychiatry and Addiction
Université de Montréal

Didier Jutras-Aswad M.D., M.Sc.
Researcher-clinician Associate Professor
Faculty of Medecine
Departement of Psychiatry and Addiction
Université de Montréal

Author:

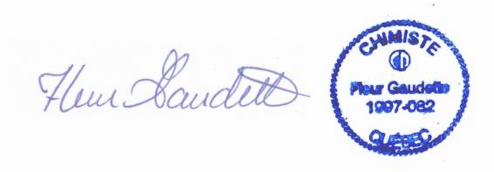

Fleur Gaudette, M.Sc. Chemist
Supervisor
Pharmacokinetics Core Facility
CRCHUM

Date: 2024-11-26

TABLE OF CONTENTS

1. Chemical Composition 22
2. Analytical Procedure 23
   2.1 Reagents 23
   2.2 Sample preparation 23
   2.3 Chromatographic conditions 23
   2.4 Mass spectrometric conditions 24
3. Chromatograms 25

LIST OF FIGURES

Figure 1: Chemical structures, formulae, and molecular weights of d_0-PEA, d_0-OEA and the internal standards (d_4-PEA & d_4-OEA) 22

Figure 2: Reconstructed ion chromatograms for d_0-PEA, d_0-OEA, d_4-PEA and d_4-OEA. (A) and (C) represent an overlay of an extracted blank surrogate matrix (red line), an LLOQ standard (blue line) and an extracted human plasma sample (green line) for d_0-PEA and d_0-OEA m/z 300 $\rightarrow$ 62 and 326 $\rightarrow$ 62, respectively. (B) and (D) represent an overlay of an extracted blank surrogate matrix (red line) and an extracted zero standard (blue line) for d_4-PEA d_4-OEA m/z 304 $\rightarrow$ 66 and 330 $\rightarrow$ 66, respectively.. 25

LIST OF TABLES

Table 1: Mass spectrometry operating conditions 24

Palmitoyl Ethanolamide and Oleoyl Ethanolamide in Human Plasma
(*Dr. François-Olivier Hébert/Dr. Didier Jutras-Aswad*)

Chemical Composition

The chemical structures, formulae, and molecular weights of palmitoyl ethanolamide (d_0-PEA), oleoyl ethanolamide (d_0-OEA) and the internal standards (d_4-PEA and d_4-OEA), are depicted in Figure 1.

Figure 3: Chemical structures, formulae, and molecular weights of d_0-PEA, d_0-OEA and the internal standards (d_4-PEA & d_4-OEA)

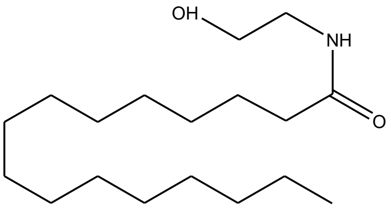

d_0-Palmitoyl Ethanolamide
d_0-PEA (target analyte)
$C_{18}H_{37}NO_2$
Mol. Wt.: 299.49

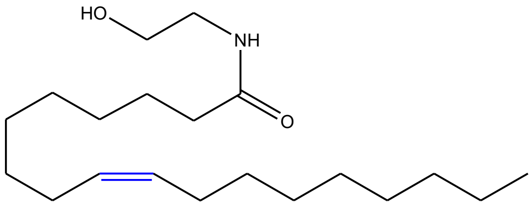

d_0-Oleoyl Ethanolamide
d_0-OEA (target analyte)
$C_{20}H_{39}NO_2$
Mol. Wt.: 325.53

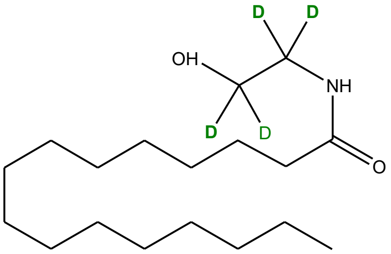

d_4-Palmitoyl Ethanolamide
d_4-PEA (internal standard)
$C_{18}H_{33}D_4NO_2$
Mol. Wt.: 303.51653

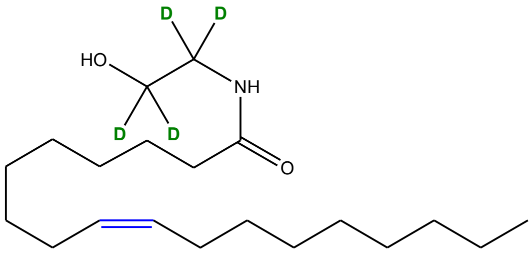

d_4-Oleoyl Ethanolamide
d_4-OEA (internal analyte)
$C_{20}H_{35}D_4NO_2$
Mol. Wt.: 329.55

Analytical Procedure

Quantitation of biomarkers by LC–MS/MS is complicated by the presence of endogenous analytes in the matrix being analyzed. Due to the endogenous nature of d_0-PEA and d_0-OEA, a surrogate matrix approach will be used. This approach involves using an authentic standard spiked into a surrogate matrix devoid of the target analyte.

Reagents

d_0-PEA, d_0-OEA, d_4-PEA, and d_4-OEA were purchased from Cayman Chemical (Ann Arbor, MI, USA) and d_4-PEA and d_4-OEA were received in ampoules as certified solutions containing 1.0 mg/mL dissolved in ethanol. Hexanes, acetonitrile, methanol and water were purchased from EMD Millipore Corporation (Burlington, MA, USA). Isopropyl alcohol (IPA) was purchased from Mat Laboratories (Québec, QC, Canada), Ethanol (EtOH) was purchase from Anachemia through VWR (Mississauga, On, Canada). Other chemicals, including, ammonium formate, and formic acid were purchased from Fisher Scientific (Fair Lawn, NJ, USA).

Sample preparation

Using a liquid-liquid extraction procedure, d_0-PEA and d_0-OEA were extracted from human plasma. Twenty five µL of internal standard solution (72.0 ng/mL of d_4-PEA and d_4-OEA in EtOH) was added to an aliquot of 300 µL of plasma in a 13 x 100 mm borosilicate screw cap tube. Two mL of extraction solvent (90:10 hexanes:IPA) was added to the sample followed by two hundred and fifty µL of water. The tube was capped and gently mixed by rotation for 10 minutes. The sample was then centrifuged at approximately 4500 *g* for 10 min at 5°C and the organic layer was transferred into a clean 13 x 100 mm borosilicate tube and evaporated to dryness at 30°C under a gentle stream of nitrogen (5-10 psi). The dried extract was re-suspended with 75 µL of reconstitution solution (50:50 methanol:H_2O) and transferred to a micro injection vial for analysis.

Chromatographic conditions

A gradient mobile phase was used with an Agilent Zorbax Eclipse Plus C18 RRHD analytical column (100 x 2.1 mm I.D., 1.8 µm) and Zorbax Eclipse Plus C18 (5.0 x 2.1 mm I.D., 1.8 µm) guard operating operating at 40°C. The initial mobile phase condition consisted of acetonitrile containing 0.1 % (v/v) formic acid and 10 mM of ammonium formate in type 1 water pH 3.0 at a

ratio of 60:40, respectively, and this ratio was maintained for 0.5 min. From 0.5 to 14 min a linear gradient was applied up to a ratio of 95:5 and maintained for 1 min. At 15.1 min, the mobile phase composition was reverted to the original conditions and the column was allowed to equilibrate for 5 min for a total run time of 20 min. The flow rate was fixed at 300 µL/min and five microliters of the extracted sample was injected.

Mass spectrometric conditions

The mass spectrometer was interfaced with the UPLC system using a pneumatic assisted heated electrospray ion source. MS detection was performed in positive ion mode, using selected reaction monitoring (SRM). In order to optimize the MS/MS parameters, standard solutions of d_0-PEA, d_0-OEA, d_4-PEA and d_4-OEA were infused into the mass spectrometer. The following parameters were obtained. Nitrogen was used for the sheath and auxiliary gases and was set at 50 and 15 arbitrary units. The HESI electrode was set to 3500V. The capillary and vaporizer temperatures were set at 350°C and 400°C, respectively. Argon was used as collision gas at a pressure of 2.5 mTorr. The precursor-ion reactions and the collision energy for PEA, OEA, d_4-PEA and d_4-OEA are in table 1. Total cycle time was set at 0.25 seconds. Peak width of Q1 and Q3 were both set at 0.7 FWHM.

Table 2: Mass spectrometry operating conditions

Compound	Polarity	Precursor (m/z)	Product (m/z)	Collision Energy (V)	RF Lens (V)
d_0-PEA	Positive	300.3	62.1	13.2	59
d_4-PEA	Positive	304.3	66.1	13.2	64
d_0-OEA	Positive	326.3	62.1	14.9	68
d_4-OEA	Positive	330.3	66.1	14.9	64

Chromatograms

Representative chromatograms obtained upon analysis of an extracted blank surrogate matrix, an extracted LLOQ standard prepared at 0.30 ng/mL for d$_0$-PEA and d$_0$-OEA in surrogate matrix and an extracted human plasma sample are shown in Figure 2.

Figure 4: **Reconstructed ion chromatograms for d$_0$-PEA, d$_0$-OEA,** d$_4$-PEA and d$_4$-OEA**. (A) and (C) represent an overlay of an extracted blank surrogate matrix (red line), an LLOQ standard (blue line) and an extracted human plasma sample (green line) for d$_0$-PEA and d$_0$-OEA m/z 300 → 62 and 326 → 62, respectively. (B) and (D) represent an overlay of an extracted blank surrogate matrix (red line) and an extracted zero standard (blue line) for** d$_4$-PEA d$_4$-OEA **m/z 304 →** **66** and **330 → 66, respectively**

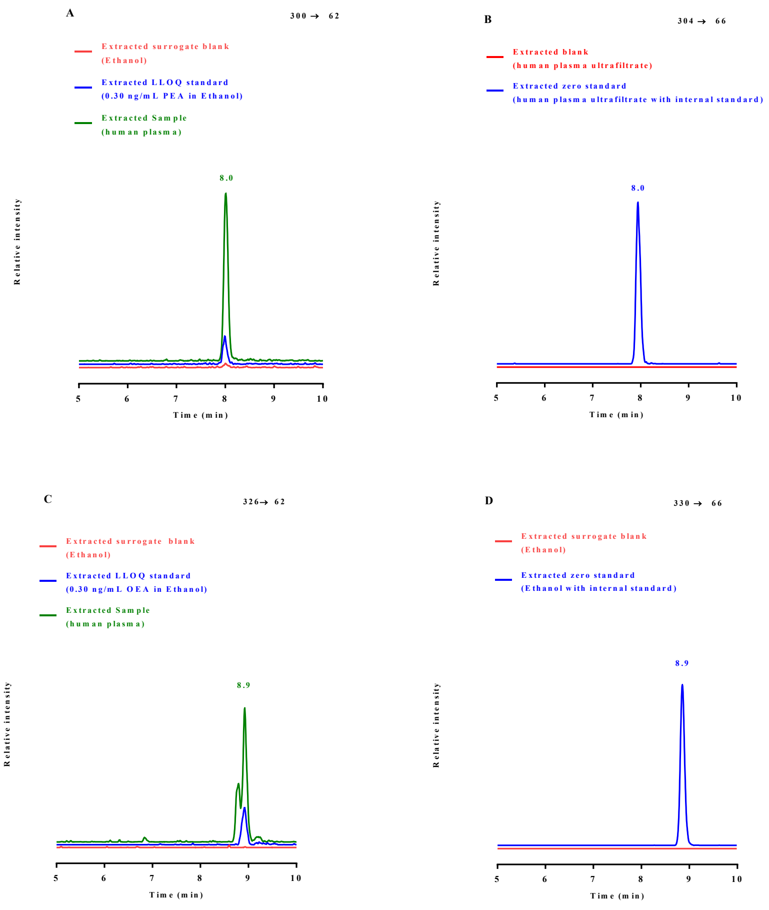

Supplement: Supplementary file 1 — Supplementary Material 1. [file 42238_2025_356_MOESM1_ESM.pdf]
